# Alterations in bacterial structure and function in seawater due to *Mytilus coruscus* farming: implications for sustainable aquaculture management

**DOI:** 10.3389/fmicb.2025.1567340

**Published:** 2025-04-03

**Authors:** Fenglin Wang, Lijia Gao, Kobi Talma, Yufeng Pan, Qi Liu, Yaodong He, Zhengwei Peng, Xiumei Zhang

**Affiliations:** ^1^Fisheries College, Zhejiang Ocean University, Zhoushan, China; ^2^School of Marine Sciences, Ningbo University, Ningbo, China; ^3^Department of Civil and Environmental Engineering, Duke University, Durham, NC, United States

**Keywords:** *Mytilus coruscus*, water, co-occurrence network, assembly mechanism, bacterial community function

## Abstract

Microorganisms are essential for maintaining the ecological balance and supporting the health of aquatic animals in aquaculture environments. This study utilized high-throughput sequencing technology to analyze the diversity, composition, co-occurrence networks, assembly mechanisms, and functional predictions of bacterial communities in seawater from both *Mytilus coruscus* aquaculture areas (AA) and non-aquaculture areas (NAA) across different seasons. The results indicated that the number of operational taxonomic units (OTUs) in the AA group was higher than the NAA group, while the Simpson index was significantly lower in the bottom water (*p* < 0.05). Additionally, the β-diversity (Bray–Curtis distance and βMNTD) was significantly reduced in the AA group compared to the NAA group (*p* < 0.05). *M. coruscus* farming influenced the relative abundance of certain genera, including *Pseudoalteromonas*, *HIMB11*, and *Clade Ia*, with the AA group exhibiting a greater number of specialist species. Co-occurrence network analysis revealed that the bacterial network in the NAA group had a higher number of nodes, edges, and modularity, whereas the AA group displayed greater closeness centrality and betweenness centrality. Following the removal of 80% of the nodes, the natural connectivity of the surface water in the AA group declined more rapidly than in the NAA group. Homogeneous selection was the primary assembly mechanism of bacterial communities in the AA group, while diffusion limitation was predominant in the NAA group. FAPROTAX functional predictions indicated the higher relative abundance of functions associated with organic matter degradation and nitrogen cycling in the AA group. These findings suggest that *M. coruscus* farming activities significantly alter the structure and function of bacterial communities in seawater, providing valuable data to support sustainable aquaculture for *M. coruscus* and optimize fisheries’ carbon sink management strategies.

## 1 Introduction

The continued rise in global carbon dioxide (CO₂) concentrations has exacerbated climate change, leading to severe environmental and social challenges (Wu et al., 2022). This phenomenon has not only resulted in increased global temperatures and more frequent extreme weather events but has also caused rising sea levels, ecological imbalances, damage to agricultural production, and threats to human health (Li L. et al., 2022; Qian et al., 2022). Reducing carbon emissions and enhancing carbon sinks are two key strategies for addressing climate change (Guo et al., 2021). The ocean serves as the largest active carbon reservoir on earth, containing 50 times more carbon than the atmosphere and playing a critical role in global climate regulation. Marine fisheries’ carbon sinks contribute to reducing atmospheric carbon dioxide concentrations directly or indirectly through fishing activities, thereby contributing positively to mitigating global climate change (Li Z. et al., 2022). Fisheries or aquaculture systems possess carbon sequestration capabilities including large-scale algae farming, bivalve farming, filter-feeding fish farming, and marine ranching (Song et al., 2024). The calcification of bivalves and their biological deposition significantly influence coastal carbon cycling. According to statistics, global bivalve aquaculture is responsible for removing approximately 7 million tons of carbon annually through shell deposition (Alonso et al., 2021). This surpasses the annual carbon sequestration of global seagrass beds, mangroves, and salt marshes, which collectively sequester around 5.3 million tons of carbon (Song et al., 2024). Furthermore, bivalves, a more environmentally friendly aquaculture species, account for 20% of global aquaculture production contributing only 7% of greenhouse gas emissions (MacLeod et al., 2020). Therefore, as a significant carbon sink fishery, bivalves play an essential role in mitigating climate change.

Microorganisms play a crucial role in maintaining the ecological balance of the ocean, promoting global biogeochemical cycles, removing marine pollutants, and sustaining the ocean’s self-purification capabilities (Urvoy et al., 2021; Zhu B. et al., 2020). As integral participants in the energy and material cycles within the marine environment, microorganisms are essential for mediating various biogeochemical processes, particularly within the carbon cycle. They have the ability to convert dissolved organic carbon (DOC) from an unstable state into refractory dissolved organic carbon (RDOC), which can be fixed and stored in the ocean for extended periods (Jiao et al., 2024), which is significant for ocean carbon storage. Consequently, the role of microorganisms in bivalve aquaculture areas is critical and cannot be overlooked. Previous studies have demonstrated that aquaculture activities can influence the organic matter content in the aquaculture environment, thereby affecting the composition and structure of microbial communities (Neissi et al., 2022; Wei et al., 2016). For example, Fang et al. (2022) found that shellfish farming in marine ranches alters the microbial structure within the aquaculture environment, while *Mytilus* farming increases microbial diversity in seawater and enhances the abundance of aerobic anoxygenic phototrophic bacteria (AAPB) populations (He et al., 2023). Co-occurrence network analysis can reveal the complex interactions within microbial communities, thereby enhancing our understanding of the dynamic changes of these communities in various environments (Guo et al., 2022). Furthermore, this analysis aids in identifying key microbial species and elucidating their significant roles in energy flow, nutrient cycling, and ecological functions. Such insights are crucial for optimizing aquaculture management, mitigating negative environmental impacts, and promoting healthy and sustainable aquaculture practices. The assembly mechanisms of microbial communities are critical factors influencing community structure and function (Bell et al., 2022; Zhu et al., 2022). Deterministic and stochastic processes represent the two primary ecological mechanisms that shape these communities. It is particularly important to gain insight into the assembly mechanisms of microbial communities, as these mechanisms and the resulting patterns of microbial diversity directly affect ecosystem functions. In aquaculture industries, examining the assembly mechanisms and ecological functions of microbial communities is of great significance for optimizing environmental management and enhancing ecological service functions.

Thick-shelled mussel (*Mytilus coruscus*) is one of the primary cultivated mussel species in China, renowned for its delicious taste, rich nutritional value, and significant economic importance (Qian et al., 2024; Zhu et al., 2023). Previous studies have indicated that the cultivation of *M. coruscus* influences the composition and structure of phytoplankton (Jiang et al., 2022), bacteria (He et al., 2023), and macrobenthos (Pei et al., 2024). However, there remains a lack of comprehensive research on the specific impacts of *M. coruscus* cultivation on bacterial community stability, assembly mechanisms, and ecological functions.

We hypothesize that *M. coruscus* can alter the composition, function, and assembly processes of bacterial communities in seawater. Consequently, this study employed high-throughput sequencing technology to analyze the bacterial communities in seawater from both *M. coruscus* aquaculture areas and non-aquaculture areas on Gouqi Island, Zhoushan City, Zhejiang Province, across different seasons. The main objectives of the study are: (1) to analyze the impact of *M. coruscus* cultivation on bacterial community diversity, composition, co-occurrence networks, and ecological functions, thereby elucidating the potential role of aquaculture activities in the microbial ecosystem of fishery carbon sinks; (2) to explore the dominant mechanisms of microbial community assembly in *M. coruscus* aquaculture areas, assessing the relative contributions of deterministic and stochastic processes to community assembly; and (3) to identify key environmental factors driving changes in the microbial communities of *M. coruscus* aquaculture areas. The findings of this research will provide a scientific basis for enhancing the sustainable cultivation of *M. coruscus* and optimizing management strategies for fishery carbon sinks.

## 2 Materials and methods

### 2.1 Study sites and sample collection

**Figure 1:**
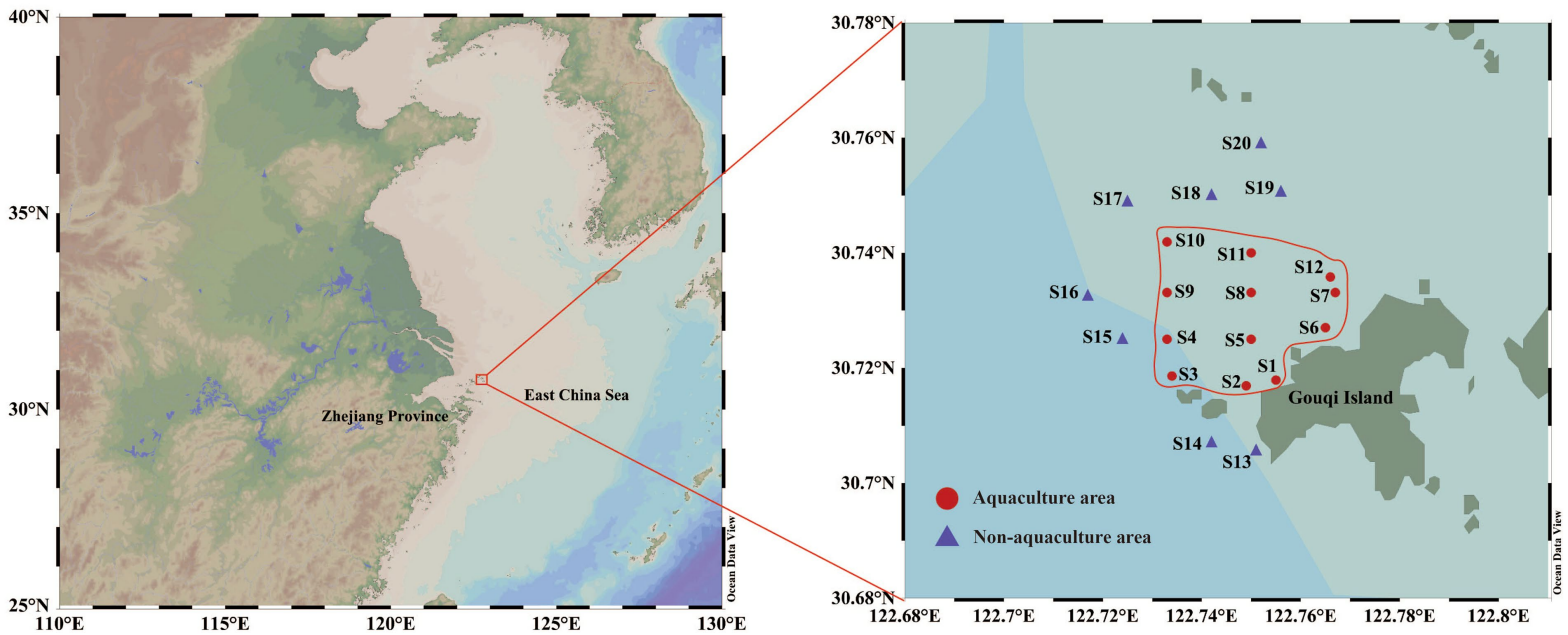
Map of sampling sites. Red circles represent *Mytilus coruscus* aquaculture areas, and blue triangles represent non-aquaculture areas.

Sea water samples were collected from the *M. coruscus* aquaculture area (AA) and non-aquaculture area (NAA) on Gouqi Island, Zhoushan City, Zhejiang Province, China, during the four seasons from April 2023 to January 2024. The specific sampling sites are illustrated in Figure 1, comprising 12 sites in the AA and eight sites in the NAA. Surface water samples (0.5–1 m below the water surface) and bottom water samples (1 m above the seabed) were collected in spring (April 2023), summer (August 2023), autumn (November 2023), and winter (January 2024). The *M. coruscus* aquaculture area on Gouqi Island is recognized as the largest mussel farming region in China, often referred to as “Mussel Town,” and is characterized by a subtropical maritime monsoon climate with distinct seasonal variations. At each site, 5 L of water was collected from both the surface and bottom layers into sterile sampling bags, stored at low temperatures, and transported to the laboratory for analysis. Three liters of water samples were filtered through a 0.22 μm mixed cellulose ester membrane. The filtered membranes were placed in 15 mL sterile centrifuge tubes and immediately frozen in liquid nitrogen for bacterial DNA extraction and high-throughput sequencing. The remaining water samples were utilized for physicochemical measurements. Water temperature (Tem), pH, salinity, and dissolved oxygen (DO) were measured *in situ* using a CTD device (SBE-25p, Sea Bird Scientific, United States). Chlorophyll a (Chl-a), total phosphorus (TP), and nutrient salts [including silicate (SiO_3_^2−^), reactive phosphate (PO_4_^3−^), nitrate-nitrogen (NO_3_^−^), nitrite-nitrogen (NO_2_^−^), and ammonium-nitrogen (NH_4_^+^)] were measured according to national standards (GB 12763.4-2007). Dissolved organic carbon (DOC) and dissolved inorganic carbon (DIC) were analyzed using a total organic carbon analyzer (Multi N/C 3100, Analytik Jena, Germany), while particulate organic carbon (POC) was measured using an elemental analyzer (FlashSmart, Thermo Scientific, United States).

### 2.2 DNA extraction, PCR amplification, and high-throughput sequencing

Total DNA from all samples was extracted using the DNeasy PowerMax Soil Kit (Qiagen, Germany) following the manufacturer’s instructions. The quality of the extracted DNA was assessed through 1% agarose gel electrophoresis, while its purity and concentration were determined using a NanoDrop2000 spectrophotometer. The V3 and V4 regions of the 16S rRNA gene were amplified with primers 338F (ACTCCTACGGGAGGCAGCAG) and 806R (GGACTACHVGGGTWTCTAAT). PCR products were extracted via 2% agarose gel electrophoresis, purified using the AxyPrep DNA Gel Extraction Kit according to the manufacturer’s protocol, and quantified using a Quantus^™^ Fluorometer (Promega, United States). The purified products were then combined in appropriate ratios to meet the sequencing volume requirements of each sample. Sequencing libraries were constructed utilizing the NEXTFLEX Rapid DNA-Seq Kit, and sequencing was conducted on an Illumina NovaSeq PE250 platform to generate raw data (Wuhan Onemore-Tech Co., Ltd.). Quality control of the raw reads was carried out using Fastp software (version 0.20.0), followed by sequence assembly with FLASH (version 1.2.7). Operational taxonomic units (OTUs) were clustered at 97% similarity using UPARSE software (version 7.1). Species classification and annotation were executed using the RDP Classifier (version 2.2) in conjunction with the Silva 16S rRNA database (version 138), applying a 70% similarity threshold.

### 2.3 Data statistical and analysis

All samples were rarefied to the lowest sequencing depth prior to subsequent analyzes. α-diversity was assessed using Chao1 and Simpson indices, alongside PCoA analysis based on Bray–Curtis distances, which were conducted using the “vegan” package in R (version 4.4.1). The β-diversity of bacterial communities was quantified using the Bray–Curtis dissimilarity metric and Beta Mean Nearest Taxon Distance (βMNTD), with the latter calculated using the “NST” package in R software (version 4.4.1). Specialist species were identified by evaluating the specificity and occupancy of the top 500 genera in both AA and NAA. A genus was classified as a specialist species if its specificity and occupancy were ≥0.7 (Dufrêne and Legendre, 1997; Gweon et al., 2021). The top 0.01% of OTUs were selected and the SparCC method was employed to conduct co-occurrence network analysis (Friedman and Alm, 2012). Following 100 bootstrap samplings utilizing the “SpiecEasi” package in R software (version 4.4.1) to obtain pseudo *p*-values, a network was constructed based on |SparCC| ≥0.7 and *p* < 0.05. Network visualization was executed using Gephi (version 0.10) (Dalcin and Jackson, 2018), and topological parameters such as modularity, clustering coefficient, average path length, density, and average degree were calculated. Within-module connectivity (Zi) and among-module connectivity (Pi) were used to evaluate key nodes in the network. Based on the values of Zi and Pi, all nodes were categorized into four classifications: peripheral nodes (Zi ≤2.5, Pi ≤0.62), connector nodes (Zi ≤2.5, Pi >0.62), module hubs (Zi >2.5, Pi ≤0.62), and network hubs (Zi >2.5, Pi >0.62). Additionally, the stability of the network was assessed by analyzing the natural connectivity of the co-occurrence network. Specifically, we examined the changes in natural connectivity following the removal of 80% of the nodes, which indirectly reflects the stability of the bacterial network (Ling et al., 2022).

The ecological functions of bacteria were predicted using the Functional Annotation of Prokaryotic Taxa (FAPROTAX). To quantify the assembly mechanisms of bacterial communities, a phylogenetic bin-based null model analysis (iCAMP) was utilized to compare the relative contributions of five community assembly processes: homogeneous selection, heterogeneous selection, dispersal limitation, homogenizing dispersal, and drift (Ning et al., 2020). Habitat niche breadth was calculated using the “spaa” package in R (version 4.4.1). Canonical Correspondence Analysis (CCA) and Redundancy Analysis (RDA) were employed to analyze the relationship between environmental factors and the bacterial community’s composition and functional composition, based on the results of detrended correspondence analysis (DCA). The variance inflation factor (VIF) for all candidate independent variables was calculated, with variables exhibiting a VIF greater than 10 being excluded to reduce the effects of multicollinearity on the model. Subsequently, significant independent variables were systematically screened through forward selection. Finally, the contribution of environmental factors to the bacterial communities’ composition and functional composition was assessed using the “rdacca.hp” package in R (version 4.4.1) (Lai et al., 2022). The Wilcoxon rank-sum test was used to compare differences between groups, with *p* < 0.05 considered statistically significant.

## 3 Results

### 3.1 Diversity of bacterial community

Venn diagram analysis revealed that OTUs in both the surface and bottom waters of the AA group exceeded that of the NAA group (Supplementary Figure S1). Specifically, the surface water of the AA group contained 2,098 unique OTUs, compared to 1,418 in the NAA group. In the bottom water, the AA group had 2,317 unique OTUs, while the NAA group had 1,392 (Supplementary Figure S1).

**Figure 2:**
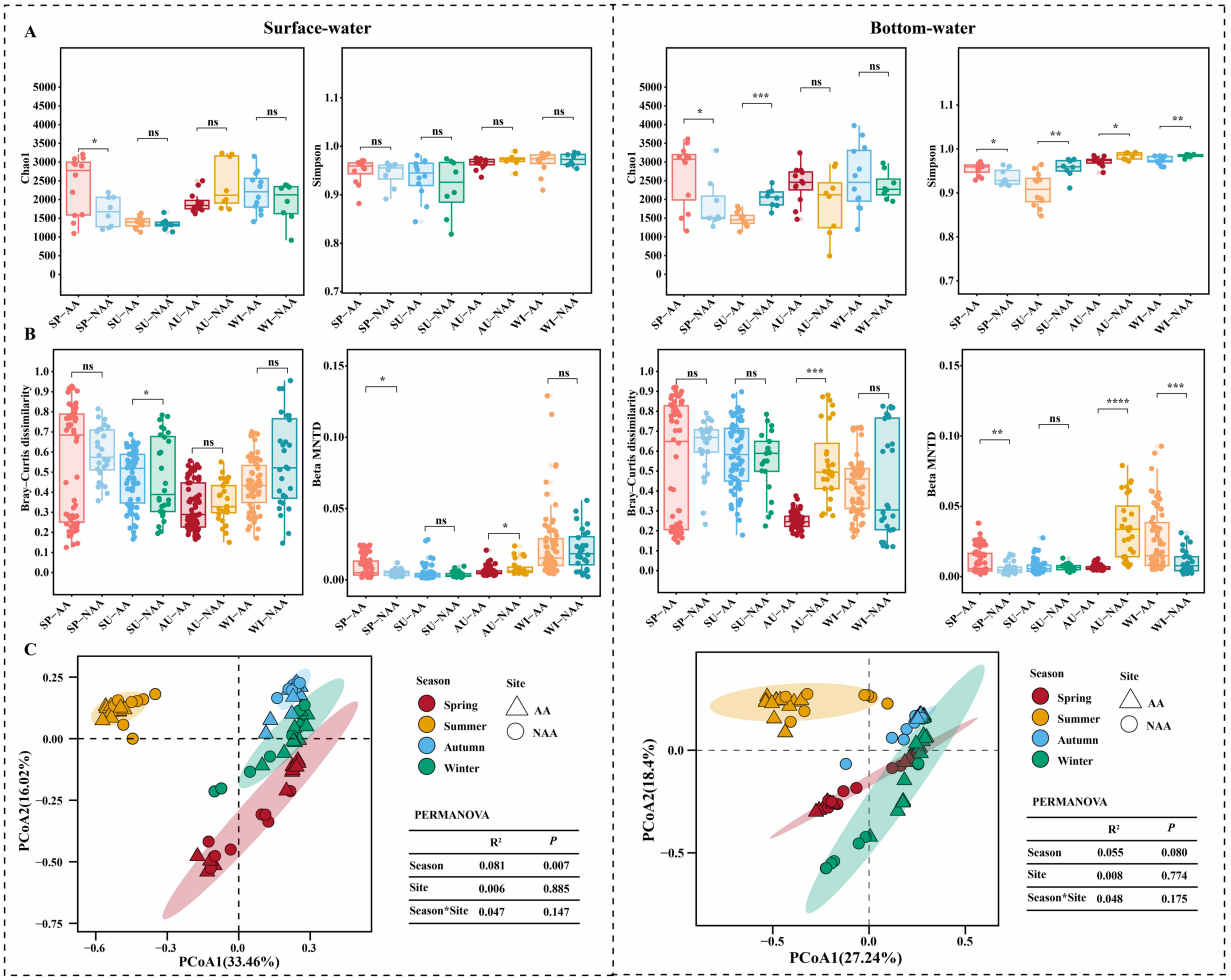
Diversity analysis of bacterial community among the surface water and bottom water during different seasons. **(A)** Comparison of alpha diversity indices (Chao1 and Simpson) between *Mytilus coruscus* aquaculture areas and non-aquaculture areas. **(B)** Comparison of beta diversity indices (Bray-Curtis’s distance and βMNTD) between *M. coruscus* aquaculture areas and non-aquaculture areas. **(C)** PCoA analysis based on the Bray-Curtis distance. “SP”, “SU”, “AU”, and “WI” represent spring, summer, autumn, and winter, respectively. “AA” and “NAA” indicate *M. coruscus* aquaculture areas and non-aquaculture areas. Wilcox test, * *P* < 0.05, ** *P* < 0.01, *** *P* < 0.001.

The results of the α-diversity analysis for surface and bottom waters across different seasons in the AA and NAA groups are presented in Figure 2A. In surface water, the Chao1 index for the AA group in spring was significantly higher than that of the NAA group (*p* < 0.05), whereas no significant differences were observed in the Simpson index in all seasons (*p* > 0.05). In bottom water, significant differences in the Chao1 index were noted between the AA and NAA groups during spring and summer (*p* < 0.05), with the Simpson index exhibiting significant differences across all seasons (*p* < 0.05). Overall, while there was no significant difference in the α-diversity of surface water between the AA and NAA groups (*p* > 0.05), the Simpson index in bottom water revealed significant variations (*p* < 0.05) (Supplementary Figure S2). The β-diversity index analysis indicated a significant decrease in β-diversity (Bray–Curtis distance and βMNTD) in both surface and bottom waters in the AA group (*p* < 0.05) (Supplementary Figure S2). In surface water, the Bray–Curtis distance for the AA group during summer was significantly higher than that of the NAA group (*p* < 0.05), and significant differences in βMNTD were observed in spring and autumn (*p* < 0.05) (Figure 2B). In bottom water, the Bray-Curtis distance for the AA group in autumn was significantly lower than that of the NAA group (*p* < 0.05), with significant differences in βMNTD noted in spring, autumn, and winter (*p* < 0.05) (Figure 2B). The results of the PCOA analysis revealed that bacterial community composition in surface water was significantly separated by season, whereas no significant separation was observed in bottom water based on season or sampling sites (Figure 2C).

### 3.2 Bacterial community composition

**Figure 3:**
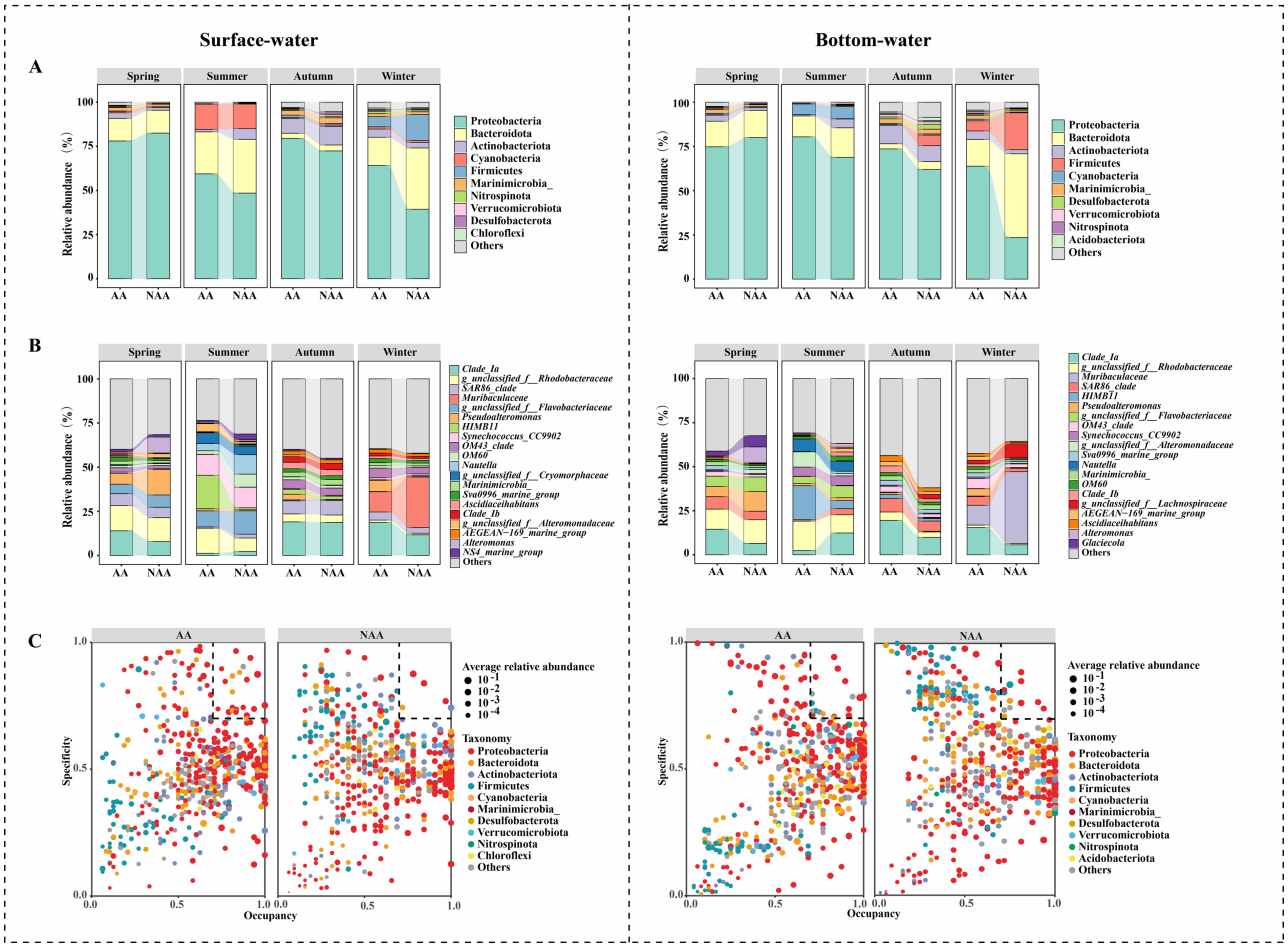
**(A)** The relative abundance of bacterial community at the phylum level in the surface water and bottom water samples. **(B)** The relative abundance of bacterial community at the genus level in the surface water and bottom water samples. **(C)** The SPEC-OCCU plots of the top 500 genera in each group. “AA” and “NAA” represent *Mytilus coruscus* aquaculture areas and non-aquaculture areas.

Proteobacteria was the predominant phylum in both surface and bottom waters across all seasons, followed by Bacteroidota and Actinobacteriota (Figure 3A). Notably, during summer, the relative abundance of Actinobacteriota in the AA group was significantly lower than that in the NAA group (*p* < 0.05). Conversely, Proteobacteria showed a higher abundance in the AA group compared to the NAA group in autumn and winter (*p* < 0.05). Additionally, in winter, the relative abundance of Bacteroidota and Firmicutes in the AA group was lower than in the NAA group (*p* < 0.05) (Supplementary Figure S3). At the genus level, the dominant genera in surface waters included *Clade Ia*, *g_unclassified_f__Rhodobacteraceae*, *SAR86_clade*, and *Muribaculaceae* (Figure 3B). In spring, the relative abundance of *Pseudoalteromonas* in the AA group was significantly lower than that in the NAA group (*p* < 0.05); however, in autumn and winter, its relative abundance was significantly higher in the AA group (*p* < 0.05). In summer, *HIMB11* showed a significantly higher relative abundance in the AA group compared to the NAA group (*p* < 0.05), while the opposite trend was observed in autumn (Supplementary Figure S4). In bottom waters, *Clade Ia*, *g_unclassified_f__Rhodobacteraceae*, *Muribaculaceae*, and *SAR86_clade* were the dominant genera (Figure 3B). During summer, the relative abundance of *Clade Ia* and *Clade Ib* in the AA group was significantly lower than in the NAA group (*p* < 0.05); and in autumn and winter, their relative abundance was significantly higher in the AA group (*p* < 0.05). Throughout summer, autumn, and winter, *HIMB11* maintains a significantly higher relative abundance in the AA group compared to the NAA group (*p* < 0.05). Furthermore, *Alteromonas* and *Muribaculaceae* exhibited high abundance in the NAA group during spring and winter, respectively (Supplementary Figure S4).

In various habitats, Proteobacteria emerged as the dominant specialist group in both surface and bottom waters, with significant variation in the specialist species between the AA group and NAA group (Figure 3C). Specifically, in surface water, the AA group hosted 22 specialist species, whereas the NAA group contained only 6. Similarly, in bottom water, the AA group was characterized by 19 specialist species, compared to 13 in the NAA group.

### 3.3 Co-occurrence networks for the bacterial communities

**Figure 4:**
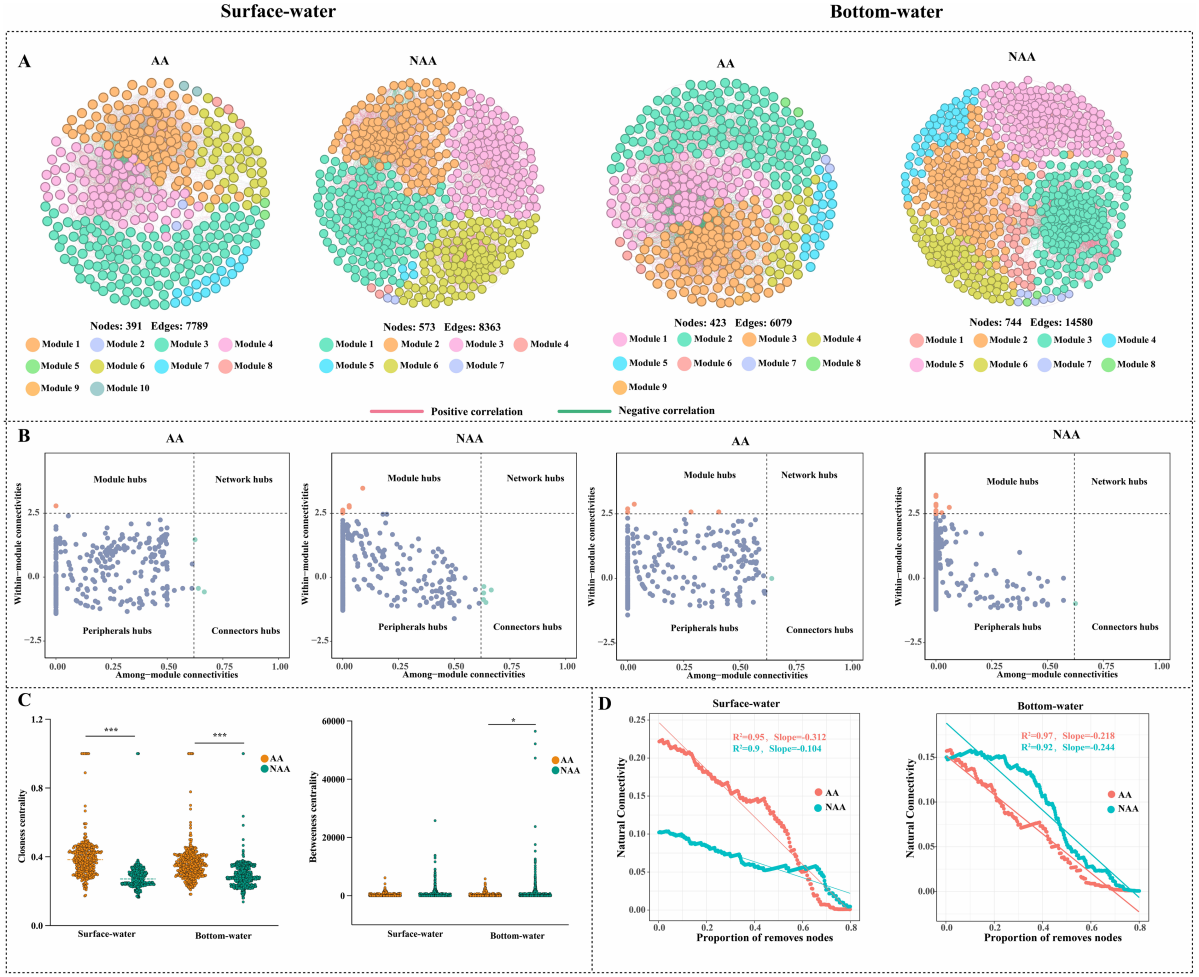
**(A)** Co-occurrence network analysis of bacterial community in surface water and bottom water. Each node represents a unique OTU and is colored by the corresponding module. A connection shows a strong Spearman correlation (|r|>0.7 and *P*<0.05). The red line indicates a positive correlation, while the green line indicates a negative relationship. **(B)** The nodes were identified as keystones within bacterial co-occurrence networks based on Zi-Pi plots. **(C)** Comparison of closeness centrality and betweenness centrality between *Mytilus coruscus* aquaculture areas and non-aquaculture areas. **(D)** The natural connectivity of bacterial networks after removing 80 % of nodes in surface water and bottom water. “AA” and “NAA” represent *M. coruscus* aquaculture areas and non-aquaculture areas.

The results of co-occurrence network analysis for surface and bottom water were illustrated in Figure 4. The findings revealed that the NAA group exhibited higher values for nodes, edges, modularity, average path length, and positive connections in both surface and bottom water compared to the AA group (Table 1). Conversely, the AA group showed more modules than the NAA group (Figure 4A). Among all networks, the phyla Proteobacteria, Bacteroidetes, and Actinobacteria demonstrated the highest levels of connectivity (Supplementary Figure S5). The results of within-module connectivity (Zi) and among-module connectivity (Pi) suggested that the NAA group possessed a greater number of potential key species than the AA group (Figure 4B). In the surface water of the AA group, there was one module hub and three connectors, whereas the NAA group had seven module hubs and five connectors. In bottom water, the AA group contains five module hubs and one connector, while the NAA group had 11 module hubs and one connector (Supplementary Table S1). Notably, the closeness centrality values for both surface water and bottom water in the AA group were significantly higher than those in the NAA group (*p* < 0.05) (Figure 4C). Additionally, the betweenness centrality of bottom water in the AA group surpassed that of the NAA group (Figure 4C). In surface water, the natural connectivity in the AA group declined at a faster rate than that in the NAA group (Figure 4D). However, no significant difference in natural connectivity was observed between the AA and NAA groups in bottom water.

**Table 1:** Topological properties of the bacterial networks.

Region	Surface-water		Bottom-water	
	AA	NAA	AA	NAA
Nodes	391	573	423	744
Edges	7,789	8,363	6,079	14,580
Positive connections	70.15%	92.98%	71.94%	95.37%
Negative connections	29.85%	7.02%	28.06%	4.63%
Network diameter	10	9	8	11
Average degree	39.841	29.19	28.742	39.194
Average path length	2.789	3.745	3.018	3.636
Density	0.102	0.051	0.068	0.053
Average clustering coefficient	0.719	0.707	0.693	0.708
Modularity	0.317	0.659	0.389	0.484

“AA” and “NAA” represent the *Mytilus coruscus* aquaculture areas and non-aquaculture areas.

### 3.4 Bacterial community assembly mechanism

**Figure 5:**
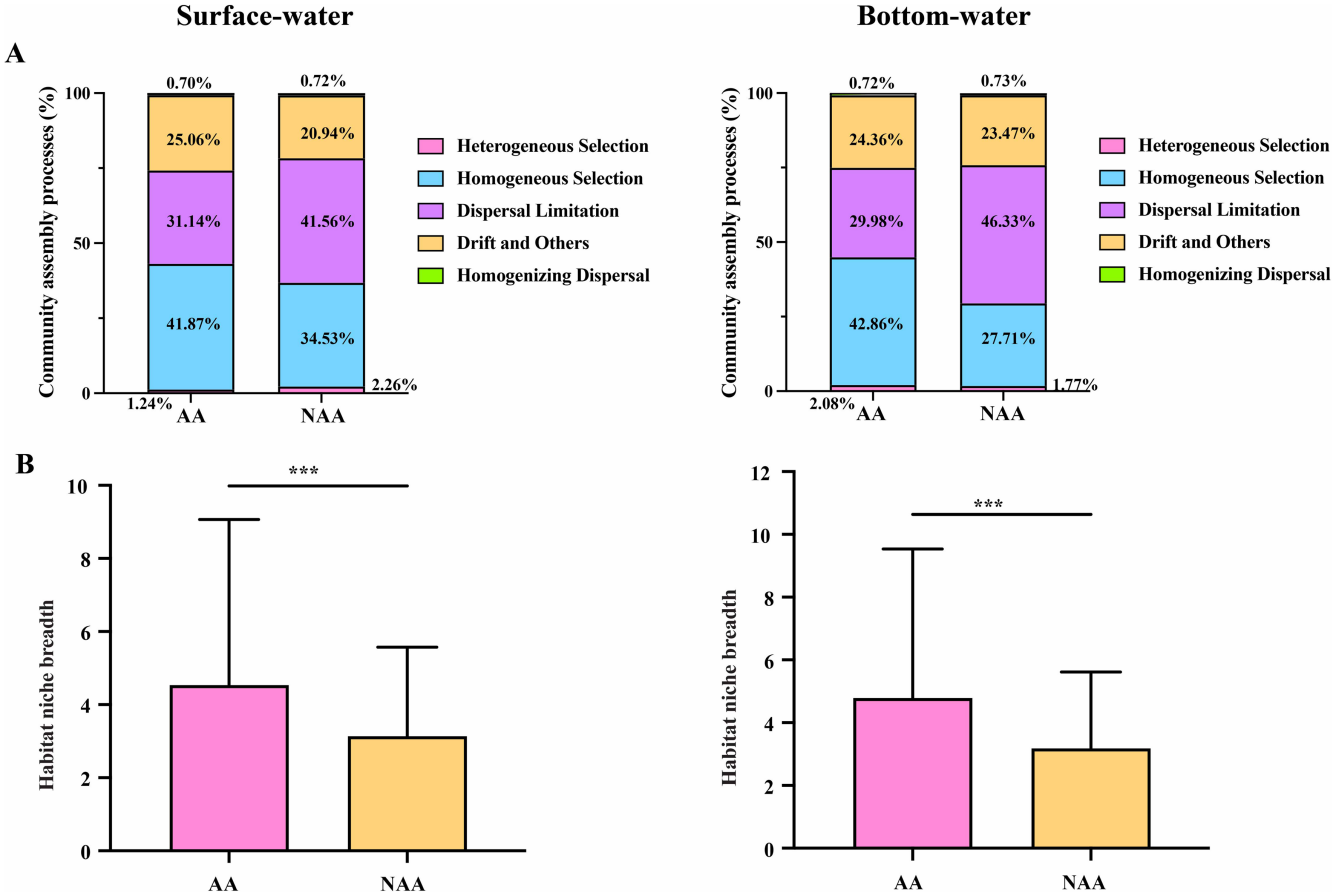
**(A)** The proportion of bacterial community assembly processes in surface water and bottom water. **(B)** Comparison of habitat niche breadth of *Mytilus coruscus* aquaculture areas and non-aquaculture areas. “AA” and “NAA” represent *M. coruscus* aquaculture areas and non-aquaculture areas. Wilcox test, *** *P* < 0.001.

The results of community assembly mechanisms revealed that stochastic processes were predominant in both AA and NAA groups (Supplementary Figure S6). Notably, the proportion of stochastic processes increased from the AA to the NAA group, while the proportion of deterministic processes decreased. This pattern suggested that aquaculture activities induced a transition in bacterial community assembly mechanisms in water, shifting from stochastic to deterministic processes. Specifically, homogeneous selection was the dominant mechanism in the AA group for both surface water (41.87%) and bottom water (42.86%), whereas dispersal limitation was more prevalent in the NAA group (surface water: 41.56%; bottom water: 46.33%) (Figure 5A). Additionally, the niche width in the AA group for both surface and bottom water was significantly greater than that observed in the NAA group (Figure 5B).

### 3.5 Prediction of bacterial functions

**Figure 6:**
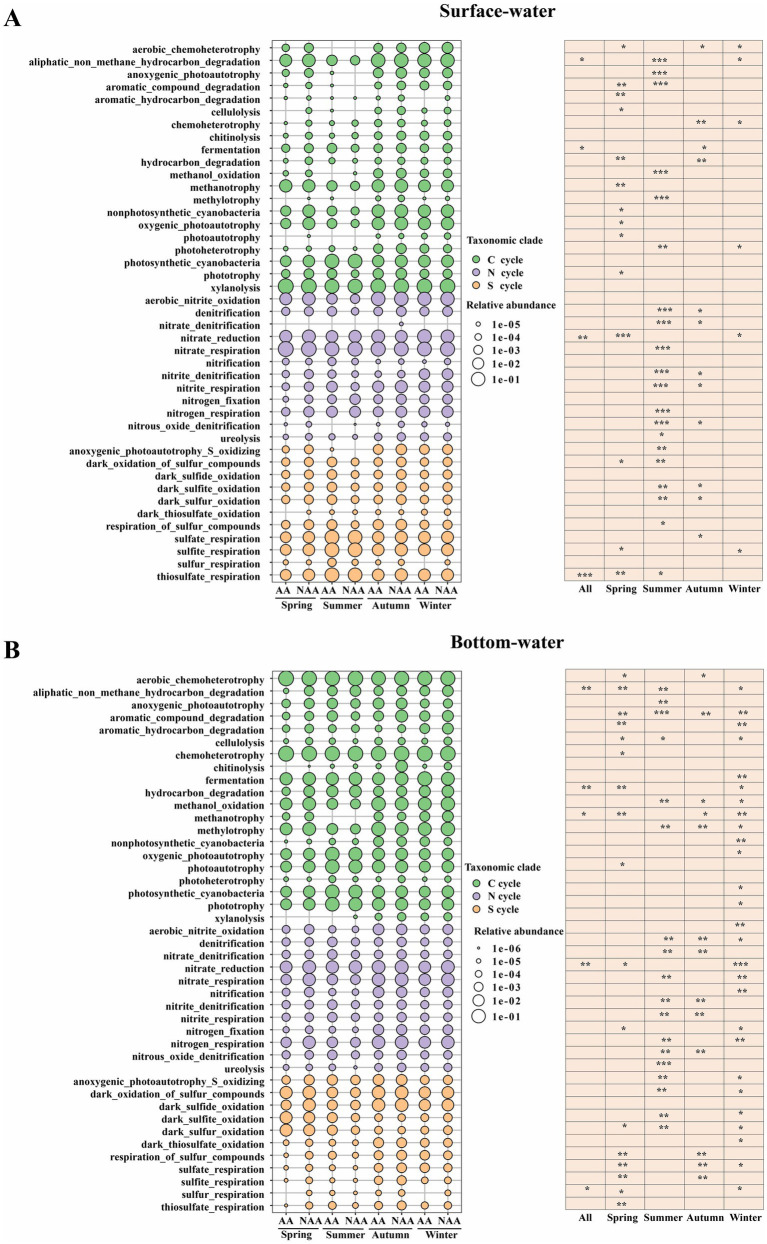
The analysis of function prediction using the FAPROTAX database in the surface water and bottom water. “AA” and “NAA” represent *Mytilus coruscus* aquaculture areas and non-aquaculture areas. Wilcox test, * *P* < 0.05, ** *P* < 0.01, *** *P* < 0.001.

Functional prediction results indicated the identification of 20 functions associated with the carbon cycle, 12 functions related to the nitrogen cycle, and 11 functions about the sulfur cycle in both surface and bottom waters. In surface water, the AA group showed significantly higher relative abundance of functions related to aliphatic non-methane hydrocarbon degradation, thiosulfate respiration, fermentation, and nitrate reduction compared to the NAA group (*p* < 0.05) (Figure 6A). Similarly, in bottom waters, the relative abundance of methanotrophy, sulfur respiration, aliphatic non-methane hydrocarbon degradation, hydrocarbon degradation, and nitrate reduction was also significantly greater in the AA group than in the NAA group (*p* < 0.05) (Figure 6B). Moreover, the most pronounced functional differences between the AA and NAA groups were observed during summer in surface waters and during winter in bottom waters, with key differences primarily related to photosynthesis, organic matter degradation, nitrification, and denitrification.

### 3.6 Physicochemical characteristics of seawater and the effect of environmental factors on bacterial communities

In surface waters, significant differences in Chl-a and SiO_3_^2−^ concentrations were observed between the AA and NAA groups across all seasons (Supplementary Table S2). The AA group exhibited higher DOC content in spring, summer, and autumn. Additionally, POC levels in the AA group were significantly elevated compared to the NAA group in summer and autumn (*p* < 0.05), while NO_3_^−^ concentrations were lower during these seasons (Supplementary Table S2). In bottom waters, SiO_3_^2−^ concentrations in the AA group were lower in spring and summer but higher in autumn and winter compared to the NAA group (Supplementary Table S3). The NO_3_^−^ levels were higher in the AA group in spring but lower in summer and autumn. DOC content remained consistently higher in the AA group across spring, summer, and autumn (Supplementary Table S3).

**Figure 7:**
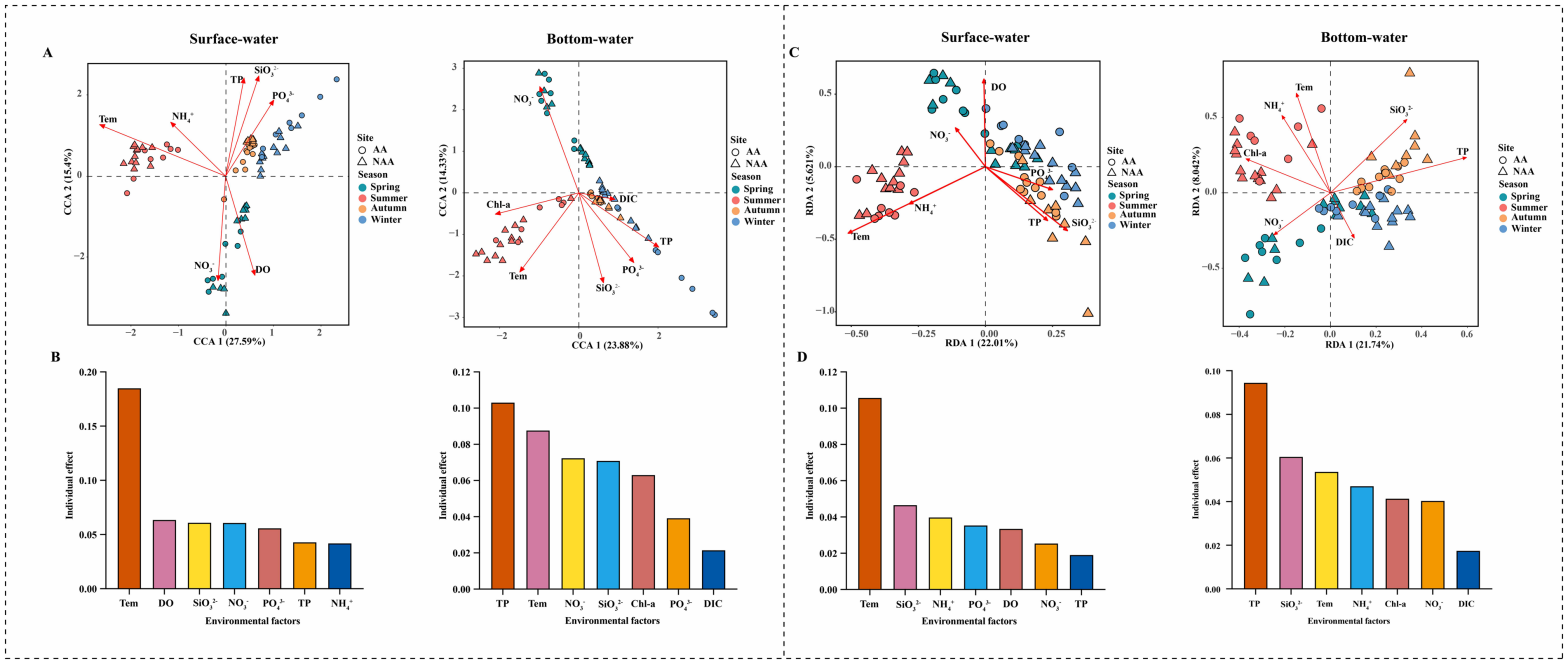
**(A)** Canonical correspondence analysis (CCA) of relationships between environmental factors and bacterial community in surface water and bottom water. **(B)** Individual effects of different environmental factors on bacterial community in surface water and bottom water. **(C)** Redundancy analysis (RDA) of relationships between environmental factors and bacterial community function in surface water and bottom water. **(D)** Individual effects of different environmental factors on bacterial community function in surface water and bottom water. “AA” and “NAA” represent *Mytilus coruscus* aquaculture areas and non-aquaculture areas.

To investigate the changes in bacterial communities in surface and bottom waters driven by environmental factors, CCA and RDA analysis were employed to assess the relationships between major physicochemical factors to both the bacterial communities’ composition and functional composition (Figures 7A,C). The results indicated that temperature was the primary factor influencing the community and functional composition of surface water, with explanatory rates of 18.5 and 10.57%, respectively (Figure 7B). Conversely, TP emerged as the predominant factor affecting the community and functional composition of bottom water, with explanatory rates of 10.31 and 9.45%, respectively (Figure 7D). Results of heatmap illustrated the correlation between differential functions and environmental factors in AA and NAA groups (Supplementary Figure S7). Notably, ammonium nitrogen exhibited a significant positive correlation with hydrocarbon degradation and aliphatic non-methane hydrocarbon degradation. Furthermore, DOC, temperature, and ammonium nitrogen in bottom water were significantly positively correlated with hydrocarbon degradation, while phosphate, TP, and POC were significantly negatively correlated with methanotrophy.

## 4 Discussion

### 4.1 *Mytilus coruscus* aquaculture affecting bacterial community diversity and composition

Microorganisms, as a vital component of ecosystems, play a crucial role in material cycling and energy flow. This study found that the *M. coruscus* aquaculture area exhibited a higher number of OTUs compared to the non-aquaculture area, suggesting that marine aquaculture activities can significantly alter the microbial community within the aquaculture water. These findings are consistent with results reported in other studies (Fang et al., 2021; Zhu W. et al., 2020). Microbial diversity in aquatic systems is an important indicator of aquaculture environmental quality, aquatic animal health, and ecological balance (Liu et al., 2024). In surface water, the Chao1 index in the aquaculture area was significantly higher during spring than in the non-aquaculture area. In bottom water, the Chao1 index in the aquaculture area was significantly elevated in spring compared to the non-aquaculture area, whereas significantly reduced in summer. This study indicated that the DOC, NO_3_^−^, and NH_4_^+^ content in the aquaculture area was significantly higher in spring than in the non-aquaculture area, while the NO_3_^−^ and NH_4_^+^ content was significantly decreased in the summer. This nutrient enrichment promotes the growth and reproduction of microorganisms, thereby enhancing species richness (Chao1 index) (Cai et al., 2014; Ma et al., 2022). Conversely, in bottom water, the Simpson index in the aquaculture area was significantly lower than that in the non-aquaculture area, with no significant difference observed in surface water. The sedimentation of organic matter may contribute to nutrient enrichment in the bottom water, leading to an increase in the relative abundance of certain species, which can dominate and diminish the evenness of bacterial communities (Simpson index). The analysis of β-diversity reveals a significant decrease (Bray–Curtis’s distance and βMNTD) in both the surface and bottom waters of the aquaculture area. This finding supports the results obtained from the Simpson index. Aquaculture activities likely contribute to the homogenization of environmental conditions within the aquaculture zone, resulting in more uniform bacterial community composition and a subsequent reduction in microbial β-diversity. Moreover, there is a notable seasonal difference in β-diversity between aquaculture and non-aquaculture areas, which may be attributed to seasonal variations in factors such as temperature and dissolved oxygen. These variations can either promote or inhibit the growth of specific microorganisms, thereby increasing community heterogeneity. In this study, PCoA results indicate that bacterial community composition is influenced by seasonal changes, particularly in surface waters. At the same time, there is no significant difference in community structure between aquaculture and non-aquaculture areas. Seasonal variation is among the most representative environmental changes, substantially impacting microbial communities (Fuhrman et al., 2015). Chen et al. (2023) found temperature as the primary driver of seasonal variation in microbial community composition and diversity in marine ranching waters. In this study, the results of CCA analysis also demonstrated that temperature is the main factor affecting the composition of bacterial communities in surface waters. Additionally, the fluidity of seawater may somewhat reduce the differences in bacterial community composition between aquaculture and non-aquaculture areas. Consequently, *M. coruscus* aquaculture enhances the bacterial species richness in both surface and bottom waters, resulting in a homogenization of the bacterial community structure and a decrease in the evenness of the bottom water. Moreover, the community diversity demonstrated notable seasonal variations.

In this study, Proteobacteria was the dominant phylum in both surface and bottom waters across all seasons, followed by Bacteroidota and Actinobacteriota. Similar findings have been observed in *Mytilus galloprovincialis* and *M. coruscus* farming (He et al., 2023; Musella et al., 2020). Proteobacteria are widely distributed in aquaculture environments (Wang et al., 2024; Zhou et al., 2021) and play a crucial role in the degradation of high-molecular-weight dissolved organic matter (Yang et al., 2022), significantly contributing to the carbon, nitrogen, and sulfur cycles in marine ecosystems. Bacteroidota is known for decomposing complex organic substances such as cellulose and pectin (Qin et al., 2021; Zhan et al., 2020), making them an important group of organic matter-degrading microbes in seawater. Actinobacteriota also contributes to organic matter decomposition and produces antibiotics or antimicrobial substances that suppress pathogens in aquaculture systems (Ghosh et al., 2023; James et al., 2023). Notably, during autumn and winter, the relative abundance of Proteobacteria in aquaculture areas was significantly higher than in non-aquaculture areas. This difference may be attributed to the elevated DOC content in aquaculture areas, which enhances the growth of Proteobacteria. Previous studies have reported that Proteobacteria are capable of removing organic matter, and an increase in DOC has been shown to promote the abundance of these bacteria (Luo et al., 2023; Rud et al., 2017). The relative abundance of Actinobacteriota in aquaculture areas was significantly lower than that in non-aquaculture areas, which may be attributed to the reduced PO_4_^3−^ and NO_3_^−^ levels in the farming areas during the summer, affecting the growth of Actinobacteriota (Wang et al., 2022; Zhang et al., 2021). At the genus level, *Clade Ia*, *g_unclassified_f__Rhodobacteraceae*, *Muribaculaceae*, and *SAR86_clade* were the predominant genera observed in both surface and bottom waters. In bottom waters, the relative abundance of *Clade Ia* and *Clade Ib* were significantly lower in aquaculture areas during the summer compared to non-aquaculture areas, yet were significantly higher in autumn and winter. *Clade Ia* and *Clade Ib*, members of the SAR11 clade, play vital roles in organic matter degradation and elemental cycling (Bolaños et al., 2022). Furthermore, during the summer, *HIMB11* exhibited a significantly higher relative abundance in aquaculture areas than non-aquaculture areas. This increase may be attributed to the abundant dissolved organic matter, including dissolved organic carbon and nitrogen sources, present in the aquaculture zones, which create favorable growth conditions for *HIMB11*. The SPEC-OCCU analysis, which evaluates the distribution of specialized and generalized microbial species across various habitats, revealed differences in microbial community structures and their adaptability to environmental conditions. In this study, aquaculture areas demonstrated a higher abundance of specialized species compared to non-aquaculture areas. This finding suggests that *M. coruscus* farming creates suitable habitats for specialized species, enhancing their richness and facilitating the decomposition of organic matter and element cycling within the aquaculture zone. Therefore, *M. coruscus* farming alters the microbial community structure of the surrounding water, and this change varies seasonally, reflecting a significant impact on material cycling and energy flow within the *M. coruscus* farming ecosystem.

### 4.2 *Mytilus coruscus* aquaculture shaping co-occurrence network structure and revealing potential bacterial interactions

Co-occurrence network analysis is crucial for elucidating the intricate relationships within microbial ecosystems and identifying key species (Faust and Raes, 2012). Aquaculture activities can significantly influence the microbial structure of the aquaculture environment, thereby affecting the complexity and stability of microbial networks. Qin et al. (2022) demonstrated that aquaculture activities can enhance the connectivity of microbial networks in sediments while diminishing it in water. Similarly, Xu et al. (2022) found that aquaculture activities reduce the network connectivity of plankton metacommunities. In this study, the network structure in non-aquaculture areas is observed to be more complex and exhibits higher connectivity compared to that in aquaculture areas, as evidenced by an increase in nodes, edges, modularity, average path length, and positive connections. The complex environment of aquaculture areas allows microbial communities to form more functional partitions, relying on specific key species to fulfill essential ecological functions. This results in lower network connectivity and a greater number of modules within aquaculture areas. Key species are defined as populations that are highly connected and centrally clustered within microbial networks, playing a vital role in sustaining community structure and function (Yao et al., 2019). The loss of these key species may precipitate network collapse. In this study, non-aquaculture areas exhibit a greater diversity of key species, which supports the finding that the network structure in these areas is more complex. Although aquaculture areas exhibit fewer key species, they can maintain ecological functions through diversified modules. For instance, *Oleispira* is capable of degrading marine hydrocarbons (Yakimov et al., 2003), while *Pseudohongiella* contributes to the degradation and transformation of organic matter within the aquaculture environment (Liao et al., 2022). Furthermore, *Muribaculaceae* effectively utilizes mucin monosaccharides and inhibits the growth of harmful bacteria (Pereira et al., 2020), thereby helping to maintain a healthy balance of gut microbiota and playing a positive role in the immune defense and barrier function of the *M. coruscus* gut. Furthermore, the closeness centrality and betweenness centrality of the network in aquaculture areas are significantly higher than those in non-aquaculture areas. This suggests that certain bacteria in aquaculture areas have become key nodes connecting other species or modules, thereby playing a vital role in material cycling and energy flow within these areas. However, the reduction or disappearance of these key species can disrupt the bacterial network in aquaculture areas. The results regarding natural connectivity indicate that after the removal of 80% of the nodes, connectivity in aquaculture areas decreases more rapidly, suggesting poorer network stability compared to non-aquaculture areas. This may be attributed to the high organic content in mussel aquaculture areas, which leads to a dependency on certain key species in these nutrient-rich environments. Consequently, when these species are disturbed, the overall stability of the network declines. Therefore, the investigation of microbial network structures and key species is essential for understanding the dynamics of microbial communities in aquaculture systems and provides theoretical support for ecological regulation and aquaculture management.

### 4.3 Null model revealing bacterial community assembly processes

Environmental heterogeneity can influence the balance between deterministic and stochastic processes, thereby affecting the distribution of microbial communities (Huber et al., 2020; Langenheder and Lindström, 2019). To investigate the impact of *M. coruscus* aquaculture activities on the assembly mechanisms of microbial communities in aquatic environments, this study employed a null model to analyze the roles of deterministic and stochastic processes. The results indicated that stochastic processes predominantly governed the bacterial community assembly mechanisms in aquaculture and non-aquaculture areas. This finding aligns with previous research highlighting the significance of stochastic processes in aquaculture water bodies (Qin et al., 2022; Wang et al., 2020). Planktonic bacteria are continuously transported by water flow and exhibit high diffusion efficiency, which contributes to the predominance of stochastic processes in these environments. However, we observed an increase in the proportion of deterministic processes in the bacterial community assembly mechanisms within the aquaculture area, accompanied by a decrease in stochastic processes. This shift suggests that aquaculture activities alter the bacterial community assembly mechanisms in water from stochastic to deterministic processes. In the aquaculture area, homogeneous selection emerged as the primary deterministic mechanism in both surface water (41.87%) and bottom water (42.86%). This indicates that *M. coruscus* aquaculture activities enhance nutrient content in the water, resulting in a more homogeneous environment that favors bacterial groups with similar niches, which play a crucial role in community construction. Zheng et al. (2023) found that in intensive farming, denitrifying communities are predominantly driven by deterministic processes, with this level of determinism increasing alongside nutrient availability. In this study, the DOC and TP content in the *M. coruscus* aquaculture area were significantly higher than those in the non-farming area. Consequently, we speculate that the increased proportion of deterministic processes in bacterial community assembly mechanisms within the aquaculture area may be attributed to the accumulation of these nutrients, which leads to the selective enrichment of bacteria with specific functions and enhances community homogeneity. When these dominant bacterial communities are disrupted, related ecological functions could collapse, severely impacting the overall stability of the ecosystem. Niche breadth is a critical factor for understanding microbial responses to environmental filtering (deterministic processes) (Liu et al., 2022). Our findings indicate that the niche breadth of water in the aquaculture area was significantly greater than that in the non-aquaculture area. *M. coruscus* farming may enhance the effects of homogeneous selection by reducing environmental heterogeneity and promoting the homogenization of farming water. This alteration could result in bacterial communities exhibiting greater similarity, thereby increasing ecological niche breadth. This finding aligns with the conclusion of this study that the assembly mechanisms of bacterial communities in aquaculture areas shift towards deterministic processes, particularly homogeneous selection. Consequently, these results not only highlight the significant impact of homogeneous selection on the assembly of bacterial communities in aquaculture areas but also provide valuable theoretical support for the sustainable development of future carbon sink fisheries.

### 4.4 Influence of *Mytilus coruscus* aquaculture on the functions of bacterial community

Microbial function prediction is essential for elucidating the roles and functions of microbial communities within ecosystems, which is vital for understanding ecosystem stability and functionality (Peng et al., 2021). In this study, the functional abundances associated with non-methane alkane degradation, thiosulfate respiration, fermentation, and nitrate reduction in surface water were significantly higher in aquaculture areas compared to non-aquaculture areas. This discrepancy may be attributed to *M. coruscus* farming, which increases the levels of organic carbon and nitrogen sources in the water, thereby enhancing the performance of specific functions within the bacterial community, particularly those related to organic matter and nitrogen cycling. In bottom water, the relative abundances of functions such as methane oxidation, sulfur respiration, non-methane alkane degradation, and nitrate reduction were also significantly higher in aquaculture areas than in non-aquaculture areas. In the *M. coruscus* farming environment, the DO levels in the bottom water are relatively low. This condition, combined with the increased organic matter content resulting from *M. coruscus* excreta and algal debris, collectively promotes the intensification of anaerobic metabolic processes, including anaerobic methane oxidation, sulfur respiration, and nitrate reduction. Correlation analysis revealed a significant positive correlation between NH_4_^+^ and both hydrocarbon degradation and aliphatic non-methane hydrocarbon degradation. This relationship may be attributed to NH_4_^+^ serving as an essential nitrogen source for the growth and metabolism of microorganisms (Daebeler et al., 2014). Furthermore, the addition of nitrogen can stimulate microbial metabolic functions and enhance the bioavailability of hydrocarbon pollutants, thereby facilitating their degradation (Liu et al., 2023; Zhang et al., 2025). In bottom water, DOC, Tem, and NH_4_^+^ exhibited significant positive correlations with hydrocarbon degradation, whereas phosphate, TP, and POC showed significant negative correlations with methanotrophy. DOC can serve as a carbon source for microbial growth, and temperature directly influences microbial metabolic rates and enzyme activity. Within an optimal temperature range, an increase in temperature can accelerate the biodegradation of hydrocarbons (Das and Chandran, 2011). The feces produced by mussel aquaculture activities not only consume substantial amounts of oxygen during decomposition but also contribute additional phosphorus, resulting in increased phosphorus concentrations in the water (Newell, 2004; Richard et al., 2006). Methane-oxidizing microorganisms typically thrive in oxygen-rich environments, and low oxygen conditions can inhibit the activity of methanotrophic bacteria. Consequently, *M. coruscus* farming can significantly alter the functional roles of bacterial communities, potentially leading to profound impacts on the stability and health of aquatic ecosystems.

Based on these research findings, we propose several specific recommendations for aquaculture management. First, reducing the stocking density of *M. coruscus* in aquaculture systems is essential for minimizing nutrient accumulation, mitigating the impact of homogenizing selection, and preserving ecosystem diversity and stability. Second, promoting integrated multi-trophic aquaculture (IMTA) can enhance the stability of the aquaculture environment and facilitate the recovery of ecosystem functions through a diverse species composition. Finally, during seasons with high nutrient content, it is advisable to strengthen nutrient management in aquaculture areas through microbial regulation to reduce their negative impact on microbial communities. These suggestions will help mitigate the potential risks of *M. coruscus* aquaculture on microbial community structure and ecosystem stability, thus providing theoretical support for the sustainable development of carbon sink fisheries.

## 5 Conclusion

**Figure 8:**
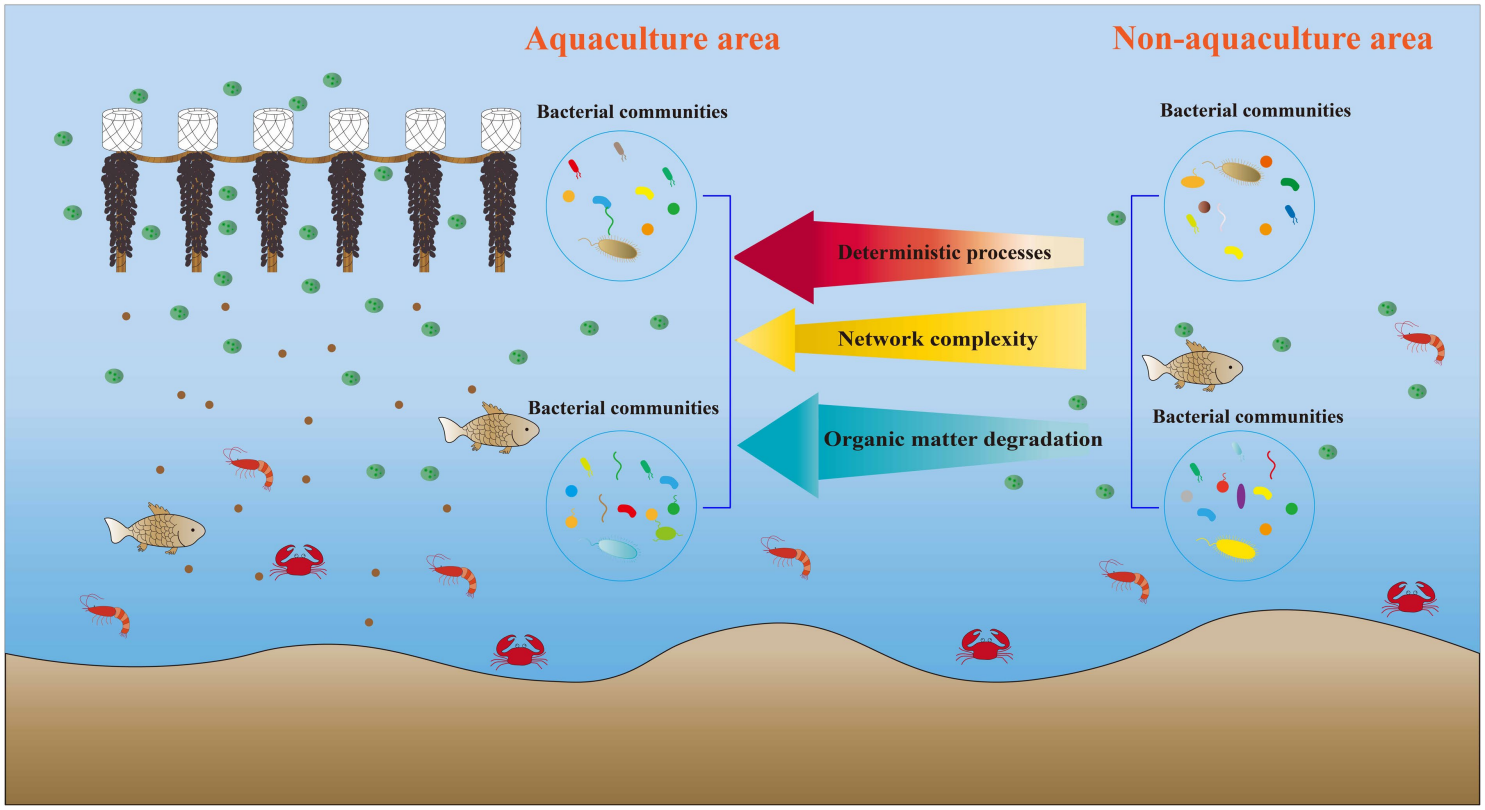
Summary of the effect of Mytilus coruscus aquaculture on seawater bacterial community.

This study investigated the impact of *M. coruscus* aquaculture on bacterial communities within the aquatic environment. The results indicated that *M. coruscus* aquaculture significantly influenced bacterial diversity and community composition. Furthermore, this aquaculture activity altered the bacterial network structure, leading to a reduction in both the complexity and stability of the network. Additionally, aquaculture activity increased the proportion of deterministic processes, particularly homogenizing selection, in the assembly of bacterial communities. Functional predictions revealed that *M. coruscus* aquaculture exhibited higher functional abundances associated with organic matter degradation and nitrogen cycling within the bacterial community (Figure 8). The primary environmental factor influencing the composition of the surface water bacterial community and its functional characteristics was Tem, whereas TP was the key factor for bottom water. Consequently, these findings provide a theoretical foundation for the sustainable development and scientific management of *M. coruscus* aquaculture.

## Data Availability

The data presented in the study are deposited in the China National Center for Bioinformation/Beijing Institute of Genomics, Chinese Academy of Sciences (https://ngdc.cncb.ac.cn/gsa) with accession number CRA023930.
