# Supplementary material for: Alterations in bacterial structure and function in seawater due to Mytilus coruscus farming: implications for sustainable aquaculture management
Source: Front Microbiol. 2025 Apr 3;16:1567340. doi: 10.3389/fmicb.2025.1567340 (PMC12005636; doi:10.3389/fmicb.2025.1567340)
Supplement: Supplementary file 1 [file Data_Sheet_1.docx]

**Alterations in bacterial structure and function in seawater due to *Mytilus coruscus* farming: Implications for sustainable aquaculture management**

Fenglin Wang ^a,b^ , Lijia Gao ^c^, Kobi Talma ^c^, Yufeng Pan ^a^, Qi Liu ^a^, Yaodong He ^a^, Zhengwei Peng ^a^, Xiumei Zhang ^a^*

^a^ Fisheries College, Zhejiang Ocean University, Zhoushan, China

^b^ School of Marine Sciences, Ningbo University, Ningbo, China

^c^ Department of Civil and Environmental Engineering, Duke University, Durham, NC, United States

*Corresponding author. Xiumei Zhang, E-mail address: xmzhang1227@163.com


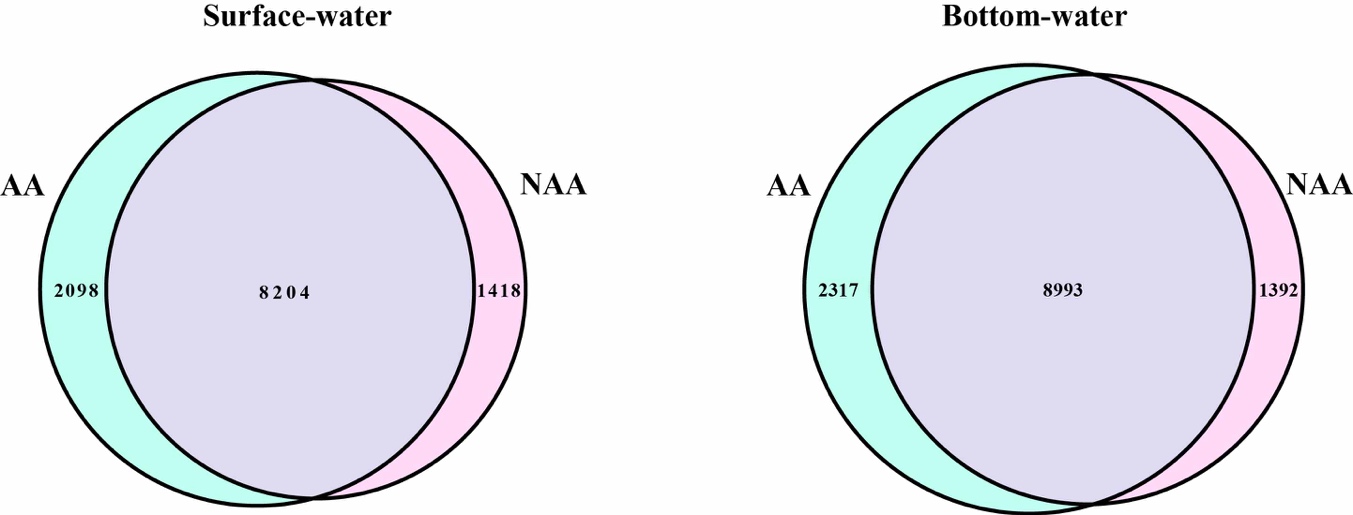


Figure S1 Venn diagram of bacterial OTUs among *Mytilus coruscus* aquaculture areas and non-aquaculture areas in the surface water and bottom water. “AA” and “NAA” represent aquaculture areas and non-aquaculture areas.


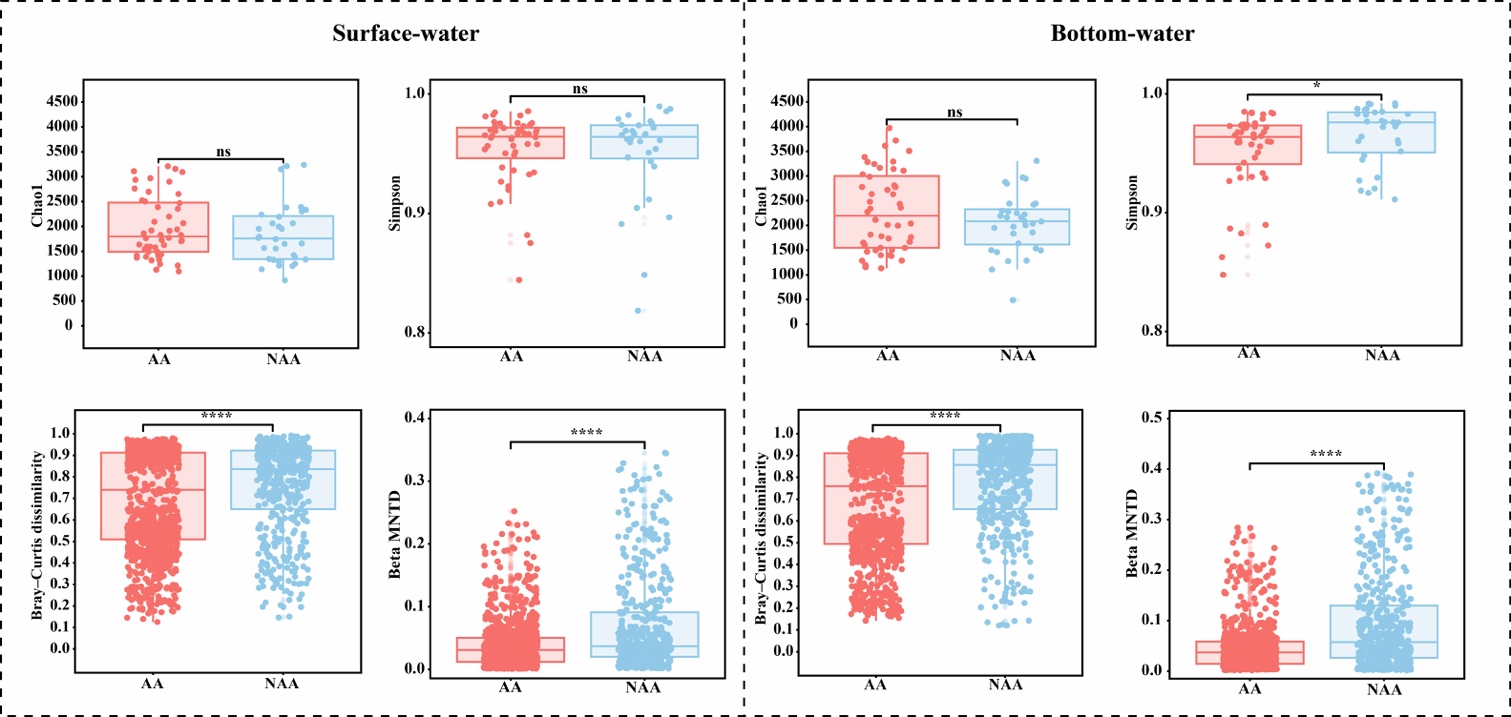


Figure S2 Comparison of alpha diversity indices (Chao1 and Simpson) and beta diversity indices (Bray-Curti’s distance and βMNTD) across *Mytilus coruscus* aquaculture areas and non-aquaculture areas. “AA” and “NAA” represent aquaculture areas and non-aquaculture areas.


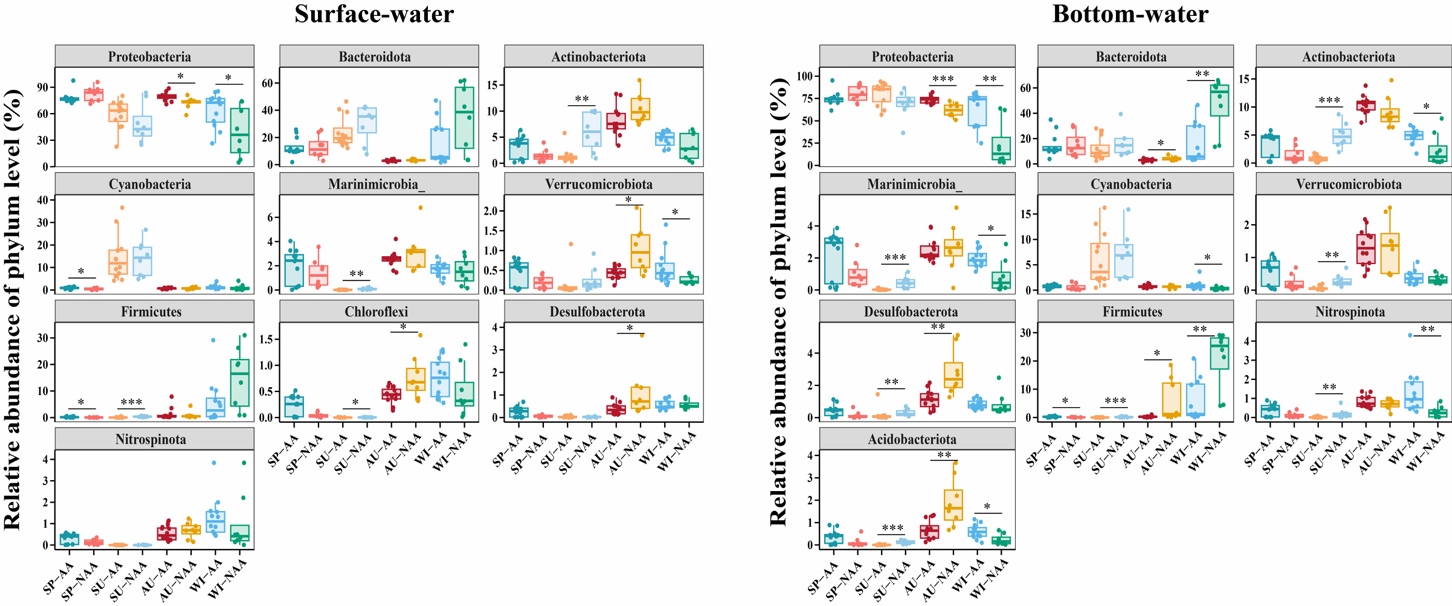


Figure S3 Differences in the relative abundance of phylum taxa among groups. “SP”, “SU”, “AU”, and “WI” represent spring, summer, autumn, and winter, respectively. “AA” and “NAA” indicate *M. coruscus* aquaculture areas and non-aquaculture areas. Wilcox test, * *P* < 0.05, ** *P* < 0.01, *** *P* < 0.001.


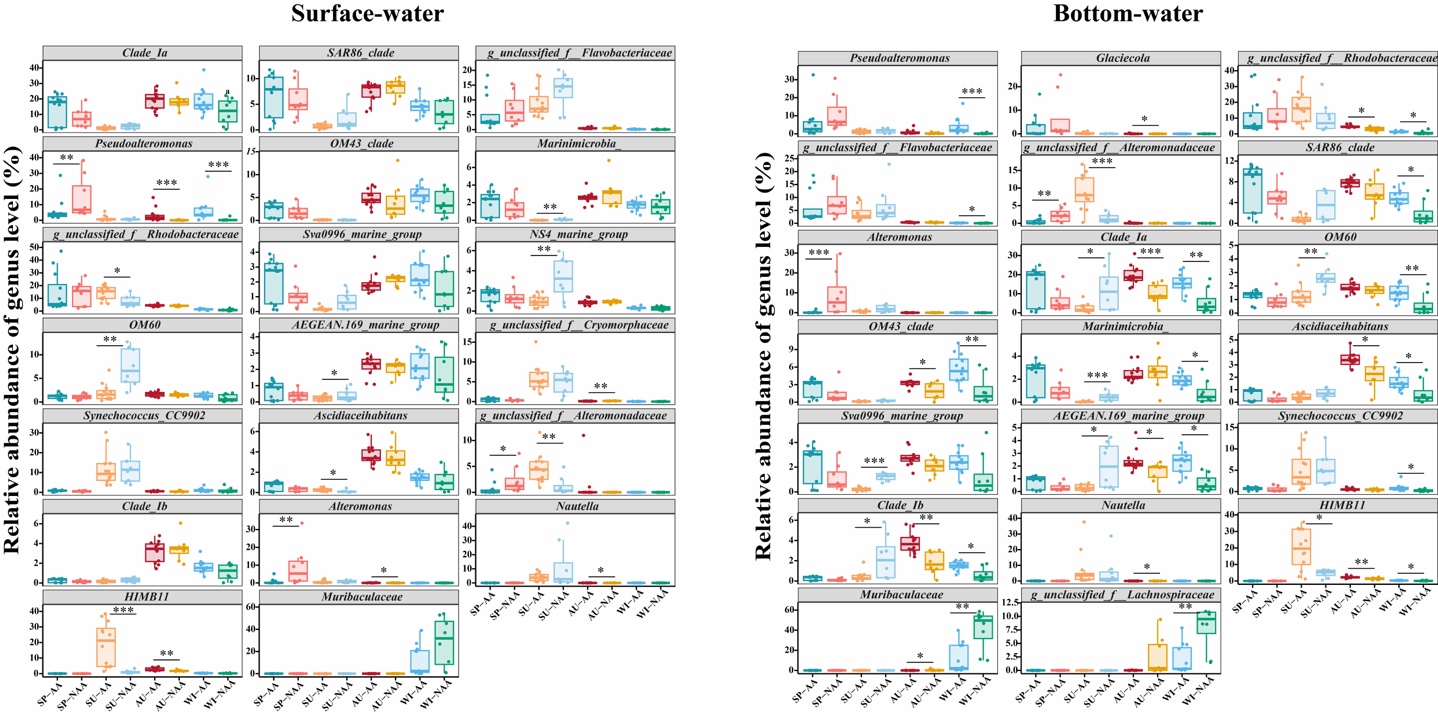


Figure S4 Differences in the relative abundance of genus taxa among groups. “SP”, “SU”, “AU”, and “WI” represent spring, summer, autumn, and winter, respectively. “AA” and “NAA” indicate *M. coruscus* aquaculture areas and non-aquaculture areas. Wilcox test, * *P* < 0.05, ** *P* < 0.01, *** *P* < 0.001.


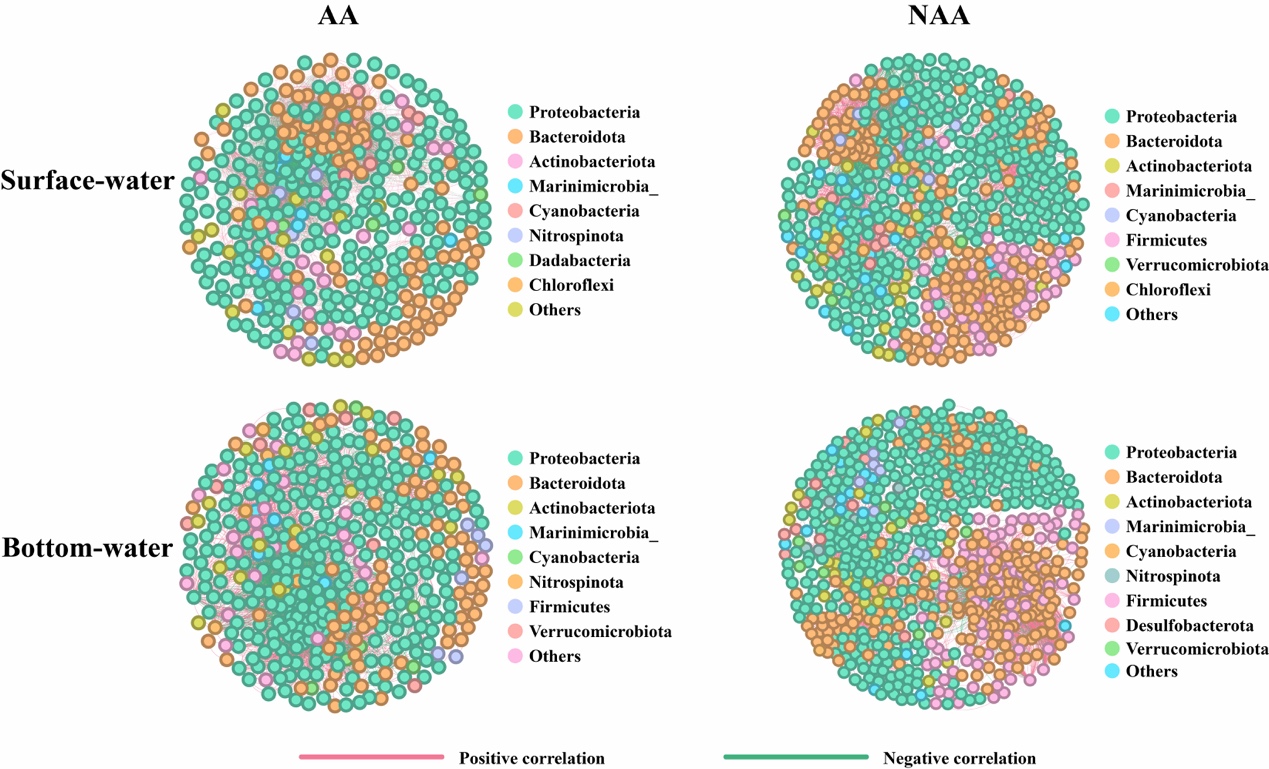


Figure S5 Co-occurrence network analysis of bacterial community in surface water and bottom water. The nodes were colored according to different phylum. Each node represents an OTU.


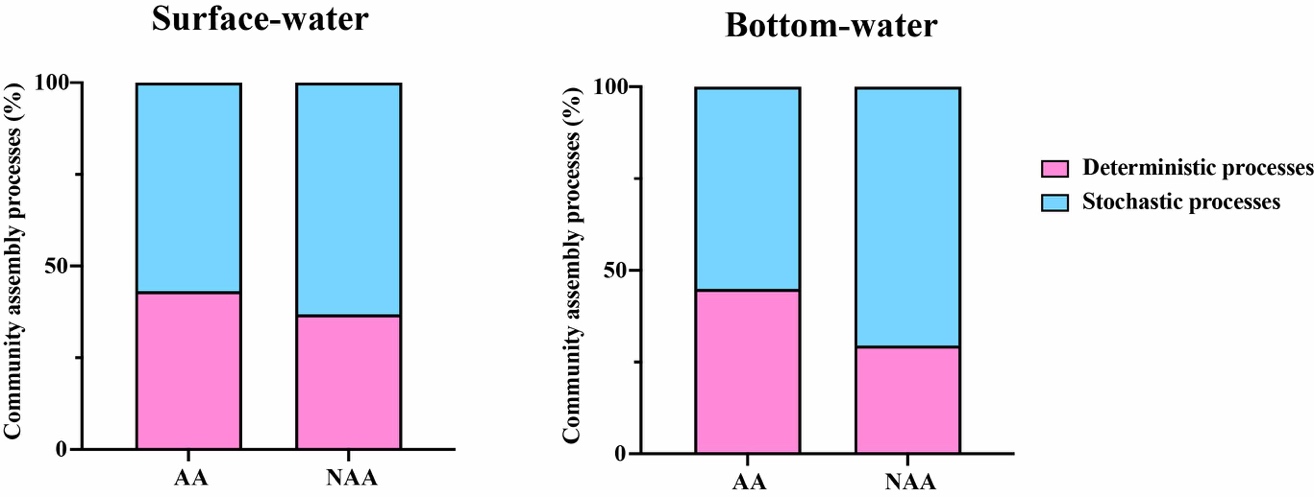


Figure S6 Relative importance of bacterial community assembly processes in surface water and bottom water.


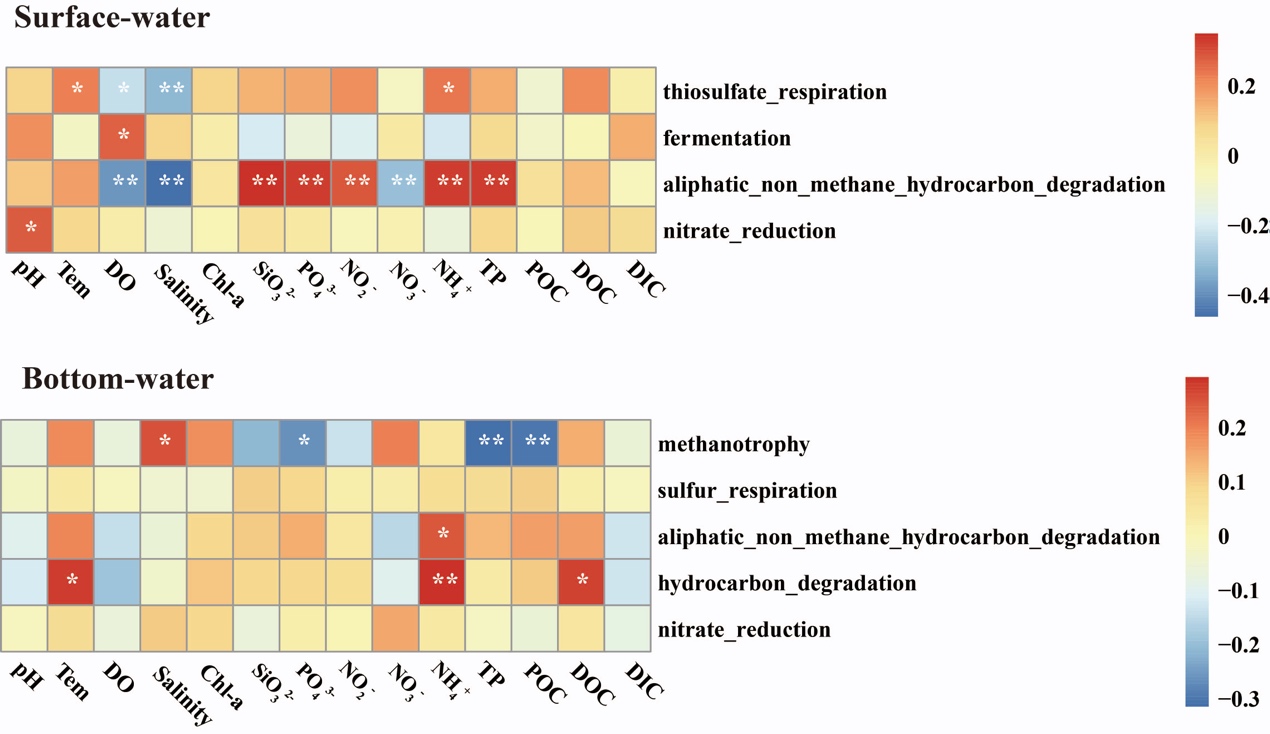


Figure S7 The heatmap of Spearman’s correlation between environmental factors and bacterial community function in surface water and bottom water.
