# Supplementary material for: Alterations in bacterial structure and function in seawater due to Mytilus coruscus farming: implications for sustainable aquaculture management
Source: Front Microbiol. 2025 Apr 3;16:1567340. doi: 10.3389/fmicb.2025.1567340 (PMC12005636; doi:10.3389/fmicb.2025.1567340)
Supplement: Supplementary file 2 [file Table_1.docx]

Table S1 Classification information of keystone taxa (Module hubs) in each bacterial network

| Site |  |  | Phylum | Genus | Within-module connectivities | Among_module connectivities | Degree | type |
| --- | --- | --- | --- | --- | --- | --- | --- | --- |
| Surface-water | AA | OTU_1647 | Proteobacteria | *Oleispira* | 1.46515887 | 0.625 | 8 | Connectors |
|  |  | OTU_6725 | Actinobacteriota | *g_unclassified_f__Ilumatobacteraceae* | -0.4395477 | 0.64 | 5 | Connectors |
|  |  | OTU_8499 | Proteobacteria | *g_unclassified_f__Thiotrichaceae* | -0.5773503 | 0.66666667 | 3 | Connectors |
|  |  | OTU_977 | Bacteroidota | *Muribaculaceae* | 2.78693206 | 0 | 14 | Module hubs |
|  | NAA | OTU_12198 | Proteobacteria | *Planktomarina* | 2.62552957 | 0 | 65 | Module hubs |
|  |  | OTU_1573 | Proteobacteria | *Colwellia* | 2.5099052 | 0 | 63 | Module hubs |
|  |  | OTU_1653 | Proteobacteria | *Marinomonas* | 2.79896612 | 0.02856543 | 69 | Module hubs |
|  |  | OTU_302 | Proteobacteria | *SAR86_clade* | -0.4963283 | 0.66597294 | 31 | Connectors |
|  |  | OTU_322 | Proteobacteria | *UBA10353_marine_group* | -0.6119527 | 0.63183673 | 35 | Connectors |
|  |  | OTU_5369 | Proteobacteria | *Oleibacter* | 2.56771739 | 0 | 64 | Module hubs |
|  |  | OTU_5931 | Proteobacteria | *Alteromonas* | 2.56771739 | 0 | 64 | Module hubs |
|  |  | OTU_785 | Bacteroidota | *NS4_marine_group* | -0.9837761 | 0.64197531 | 9 | Connectors |
|  |  | OTU_834 | Bacteroidota | *Polaribacter* | 2.74115393 | 0.02897924 | 68 | Module hubs |
|  |  | OTU_8476 | Proteobacteria | *g_unclassified_f__Rhodobacteraceae* | 3.4797756 | 0.08950784 | 86 | Module hubs |
|  |  | OTU_8490 | Proteobacteria | *HIMB11* | -0.3622689 | 0.6326743 | 31 | Connectors |
|  |  | OTU_9276 | Proteobacteria | *g_unclassified_f__Rhodobacteraceae* | -0.8707748 | 0.63111111 | 15 | Connectors |
| Bottom-water | AA | OTU_1586 | Proteobacteria | *Pseudohongiella* | 0 | 0.64197531 | 9 | Connectors |
|  |  | OTU_2359 | Bacteroidota | *Muribaculaceae* | 2.56282797 | 0 | 15 | Module hubs |
|  |  | OTU_297 | Proteobacteria | *OM43_clade* | 2.57342114 | 0.28222931 | 73 | Module hubs |
|  |  | OTU_308 | Proteobacteria | *Pseudohongiella* | 2.57342114 | 0.40600833 | 82 | Module hubs |
|  |  | OTU_322 | Proteobacteria | *UBA10353_marine_group* | 2.87452811 | 0.02940521 | 67 | Module hubs |
|  |  | OTU_8476 | Proteobacteria | *g_unclassified_f__Rhodobacteraceae* | 2.69386393 | 0 | 63 | Module hubs |
|  | NAA | OTU_12198 | Proteobacteria | *Planktomarina* | 2.53776786 | 0.02596953 | 76 | Module hubs |
|  |  | OTU_12515 | Proteobacteria | *g_unclassified_f__Rhodobacteraceae* | 3.22141633 | 0 | 89 | Module hubs |
|  |  | OTU_302 | Proteobacteria | *SAR86_clade* | 2.86857251 | 0 | 65 | Module hubs |
|  |  | OTU_5369 | Proteobacteria | *Oleibacter* | 3.1725843 | 0 | 88 | Module hubs |
|  |  | OTU_6665 | Actinobacteriota | *Candidatus_Actinomarina* | 2.62405717 | 0 | 61 | Module hubs |
|  |  | OTU_765 | Bacteroidota | *g_unclassified_f__Flavobacteriaceae* | 2.62405717 | 0 | 61 | Module hubs |
|  |  | OTU_771 | Bacteroidota | *NS5_marine_group* | 2.74631484 | 0.05964497 | 65 | Module hubs |
|  |  | OTU_825 | Bacteroidota | *NS5_marine_group* | -0.9825442 | 0.625 | 4 | Connectors |
|  |  | OTU_834 | Bacteroidota | *Polaribacter* | 2.58659989 | 0 | 76 | Module hubs |
|  |  | OTU_8476 | Proteobacteria | *g_unclassified_f__Rhodobacteraceae* | 2.56292833 | 0 | 60 | Module hubs |
|  |  | OTU_8511 | Proteobacteria | *Ascidiaceihabitans* | 2.5017995 | 0 | 59 | Module hubs |
|  |  | OTU_8593 | Proteobacteria | *Clade_Ib* | 2.80744368 | 0 | 64 | Module hubs |

Note: “AA” and “NAA” represent the *Mytilus coruscus* aquaculture areas and non-aquaculture areas.

Table S2 Difference in environmental factors between aquaculture areas and non-aquaculture areas in surface water during different seasons.

| Group | Spring | | Summer | | Autumn | | Winter | |
| --- | --- | --- | --- | --- | --- | --- | --- | --- |
|  | AA | NAA | AA | NAA | AA | NAA | AA | NAA |
| Tem | 12.78±0.05 | 12.78±0.06 | 26.31±0.60 | 25.93±0.99 | 18.41±0.45 | 18.75±0.67 | 12.34±0.18 | 12.43±0.36 |
| pH | 8.08±0.06 | 8.07±0.05 | 8.02±0.06 | 8.04±0.11 | 8.13±0.16 | 8.23±0.12 | 8.15±0.10 | 8.12±0.12 |
| DO | 10.28±0.53 | 9.95±0.42 | 7.03±0.66 | 6.82±1.07 | 5.89±0.23^a^ | 6.34±0.40^b^ | 8.37±0.16 | 8.28±0.14 |
| Salinity | 31.2±0.13 | 30.97±0.22 | 28.66±0.61 | 27.87±1.01 | 29.23±0.68 | 29.59±0.87 | 30.52±0.58 | 29.84±1.08 |
| Chl-a | 2.21±0.36^a^ | 1.75±0.24^b^ | 3.84±0.31^a^ | 2.92±0.41^b^ | 2.48±0.40^a^ | 1.77±0.43^b^ | 0.63±0.09^a^ | 0.89±0.11^b^ |
| SiO_3_^2-^ | 0.28±0.03^a^ | 0.32±0.02^b^ | 0.56±0.03^a^ | 0.62±0.05^b^ | 0.95±0.08^a^ | 0.71±0.14^b^ | 0.76±0.07^a^ | 0.65±0.06^b^ |
| PO_4_^3-^ | 0.01±0.00 | 0.01±0.00 | 0.01±0.00^a^ | 0.02±0.01^b^ | 0.01±0.00 | 0.01±0.00 | 0.02±0.00 | 0.02±0.00 |
| NO_2_^-^ | 0.01±0.00 | 0.01±0.00 | 0.01±0.00^a^ | 0.02±0.00^b^ | 0.01±0.00 | 0.01±0.00 | 0.01±0.00 | 0.01±0.00 |
| NO_3_^-^ | 0.15±0.01^a^ | 0.01±0.01^b^ | 0.04±0.01^a^ | 0.08±0.01^b^ | 0.04±0.01^a^ | 0.08±0.01^b^ | 0.03±0.01 | 0.02±0.00 |
| NH_4_^+^ | 0.02±0.00 | 0.02±0.00 | 0.03±0.00^a^ | 0.06±0.01^b^ | 0.03±0.01^a^ | 0.02±0.01^b^ | 0.02±0.01^a^ | 0.03±0.01^b^ |
| TP | 0.02±0.00^a^ | 0.01±0.00^b^ | 0.04±0.01^a^ | 0.02±0.01^b^ | 0.05±0.01 | 0.04±0.00 | 0.04±0.00 | 0.04±0.00 |
| POC | 0.17±0.04^a^ | 0.24±0.05^b^ | 0.24±0.05^a^ | 0.13±0.04^b^ | 0.47±0.12^a^ | 0.25±0.06^b^ | 0.23±0.05 | 0.30±0.10 |
| DOC | 7.72±0.57^a^ | 6.61±0.36^b^ | 9.01±0.57^a^ | 8.11±0.23^b^ | 8.76±0.72^a^ | 7.48±0.50^b^ | 6.28±0.38 | 6.51±0.37 |
| DIC | 24.00±1.68 | 24.48±1.91 | 22.43±0.50 | 22.27±0.60 | 22.11±0.34^a^ | 24.00±0.77^b^ | 25.30±0.29 | 25.02±0.69 |

Note: “AA” and “NAA” represent the *Mytilus coruscus* aquaculture areas and non-aquaculture areas.

Table S3 Difference in environmental factors between aquaculture areas and non-aquaculture areas in bottom water during different seasons

| Group | Spring | | Summer | | Autumn | | Winter | |
| --- | --- | --- | --- | --- | --- | --- | --- | --- |
|  | AA | NAA | AA | NAA | AA | NAA | AA | NAA |
| Tem | 12.13±0.10 | 12.06±0.08 | 21.92±0.52 | 21.52±0.43 | 19.31±0.35 | 19.74±0.49 | 12.85±0.27 | 13.18±0.45 |
| pH | 8.03±0.04 | 7.97±0.04 | 7.71±0.03 | 7.69±0.03 | 7.93±0.17 | 8.06±0.13 | 8.01±0.14 | 8.02±0.10 |
| DO | 8.67±0.63 | 7.99±0.04 | 2.72±0.50^a^ | 2.18±0.26^b^ | 5.40±0.28^a^ | 5.90±0.24^b^ | 8.11±0.15^a^ | 7.76±0.21^b^ |
| Salinity | 31.88±0.55 | 32.14±0.56 | 32.72±0.36 | 32.92±0.68 | 30.70±0.53 | 31.06±0.38 | 31.40±0.47 | 31.00±0.98 |
| Chl-a | 1.25±0.27 | 1.03±0.23 | 2.19±0.27^a^ | 1.24±0.26^b^ | 1.69±0.35^a^ | 0.80±0.27^b^ | 0.63±0.21 | 0.55±0.19 |
| SiO_3_^2-^ | 0.34±0.03^a^ | 0.38±0.03^b^ | 0.66±0.05^a^ | 0.74±0.03^b^ | 0.84±0.06^a^ | 0.75±0.05^b^ | 0.73±0.05^a^ | 0.63±0.09^b^ |
| PO_4_^3-^ | 0.01±0.00 | 0.01±0.00 | 0.01±0.02^a^ | 0.02±0.00^b^ | 0.01±0.00 | 0.01±0.00 | 0.03±0.00^a^ | 0.03±0.00^b^ |
| NO_2_^-^ | 0.01±0.00 | 0.01±0.00 | 0.01±0.00^a^ | 0.01±0.00^b^ | 0.02±0.00^a^ | 0.01±0.00^b^ | 0.01±0.00^a^ | 0.01±0.00^b^ |
| NO_3_^-^ | 0.13±0.01^a^ | 0.10±0.01^b^ | 0.04±0.00^a^ | 0.06±0.00^b^ | 0.04±0.01^a^ | 0.06±0.01^b^ | 0.02±0.00 | 0.02±0.01 |
| NH_4_^+^ | 0.02±0.00^a^ | 0.01±0.00^b^ | 0.05±0.01^a^ | 0.06±0.01^b^ | 0.03±0.00 | 0.03±0.00 | 0.03±0.01^a^ | 0.02±0.00^b^ |
| TP | 0.02±0.00^a^ | 0.01±0.00^b^ | 0.02±0.00 | 0.02±0.01 | 0.04±0.00 | 0.04±0.01 | 0.04±0.00 | 0.03±0.01 |
| POC | 0.19±0.05 | 0.15±0.04 | 0.15±0.04 | 0.16±0.06 | 0.25±0.04 | 0.23±0.03 | 0.24±0.04 | 0.24±0.04 |
| DOC | 7.52±0.42^a^ | 6.40±0.25^b^ | 8.97±0.54^a^ | 8.31±0.39^b^ | 8.39±0.54^a^ | 6.91±0.48^b^ | 6.32±0.40 | 6.16±0.42 |
| DIC | 24.64±0.77^a^ | 23.15±0.76^b^ | 23.41±0.86 | 23.46±0.70 | 22.16±0.56^a^ | 24.02±0.73^b^ | 25.44±0.38^a^ | 25.03±0.41^b^ |

Note: “AA” and “NAA” represent the *Mytilus coruscus* aquaculture areas and non-aquaculture areas.
